# The Chemokine CXCL8 in Carcinogenesis and Drug Response

**DOI:** 10.1155/2013/859154

**Published:** 2013-10-09

**Authors:** Dominique Gales, Clarence Clark, Upender Manne, Temesgen Samuel

**Affiliations:** ^1^Center for Cancer Research and Department of Pathobiology, Tuskegee University, 1200 Old Montgomery Road, School of Veterinary Medicine, Tuskegee, AL 36830, USA; ^2^Morehouse School of Medicine, 720 Westview Drive, S.W., Atlanta, GA 30310, USA; ^3^Department of Pathology, and Comprehensive Cancer Center, University of Alabama, Birmingham, 1720 Second Avenue South, AL 35294, USA

## Abstract

Although the functions of chemokines in the regulation of immune processes have been studied in some detail, the role of these biomolecules in cancer is not fully understood. Chemokines mediate migration of immune cells and other functions related to immunity. They are also involved in oncogenesis and in tumor progression, invasion, and metastasis through mechanisms similar to their roles in immune functions. Various chemokines also promote cell proliferation and resistance to apoptosis of stressed cells. Consequently, chemokines and their receptors present potential therapeutic targets for anticancer drugs. The chemokine CXCL8, also known as interleukin-8 (IL8), is a proinflammatory molecule that has functions within the tumor microenvironment. Due to its potent angiogenic effects and the activity of the chemokine and its receptors in the promotion of invasion and metastasis, CXCL8 and its receptors are now considered as attractive targets for cancer therapy. This review relates the current understanding of the regulation, signaling, and functions of CXCL8 that contribute to tumor growth and metastasis, and of its role in drug response.

## 1. Introduction

Chemokines, a family of structurally related 8–10 kDa protein molecules, are secreted in diverse tissue environments and are characterized by their involvement in the regulation of hematopoietic cells and inflammatory processes [1, 2]. To date, more than 50 chemokines, which signal through about 20 G-protein-coupled receptors, have been identified [1, 3, 4]. 

Chemokines are divided into 4 subfamilies (C, CC, CXC, and CX3C), based on their primary structure or function. The structural basis for their classification is the location of the main cysteine residues in their N terminal regions [5–7]. Functionally, chemokines are categorized as “inflammatory” or “homeostatic.” Inflammatory chemokines are released primarily in response to infections; homeostatic chemokines are constitutively secreted at specific sites in the body, such as in the lymphoid organs, where they serve to attract cells that express cognate receptors [8–12].

 Although genetic alterations determine the cell of origin of cancer, microenvironmental factors are now known to control the development and progression of the malignant process; hence, these factors are included as a new paradigm in Hallmarks of Cancer [13]. In addition to cancer cells, the tumor microenvironment includes fibroblasts, endothelial cells, macrophages, lymphocytes, neutrophils, and mast cells, all of which respond to various stimuli and communicate through contact and by secreted mediators. 

## 2. Chemokines and the “Inflamed” Tumor Microenvironment 

As in infection-associated inflammatory processes, chemokines are the driving forces for immune cell infiltration into tumor tissues [14, 15]. This raises a question of whether the increased production of chemokines in tumors is an indication of progression to malignancy or a defensive reaction to an “inflammatory state” created by malignant cells. The link between an inflammatory state and cancer can be viewed from a cell-intrinsic perspective, for which genetic lesions (e.g., Ras or RET activation) initiate inflammatory signaling and an “inflamed” microenvironment; and from an extrinsic perspective, for which infection and subsequent chronic inflammation drive mechanisms that result in oncogenesis [16–18]. In either case, cancers associated with inflammation are generally aggressive.

 Through cellular and acellular components, chemokines, growth factors, and growth factor receptors, there is a complex intratumoral communication network that results in a microenvironment resembling a site of microbial infection. While the need for an effective immune response to an infection by a pathogen is evident, the infiltration of immune cells into an organ (or tumor) [19] could be considered as a reaction analogous to that of a local infection. Such infiltrating immune cells may have a role in the earliest stages of oncogenesis before the growing tumor requires a vascular supply, and when clearance of dead cells and debris is needed. Since immune cells recognize and remove cells with altered expression of cell surface markers [20], the complex signaling network in the microenvironment could lead either to progression or rejection of the tumor. In such a scenario, chemokines are considered to have dual functions, on one hand by supporting the immune system to coordinate antitumor immunity and, on the other hand, by facilitating the release of factors that promote angiogenesis and the recruitment of immunoregulatory cells, including myeloid-derived suppressor cells. The latter process supports tumor development rather than rejection [21, 22]. 

Many chemokines and their receptors expressed by both epithelial and stromal cells are associated with cancer progression [19, 23, 24]. Chemokines that contribute to immune infiltration into tumor sites and tumor growth include the growth-related (GRO) family of chemokines (CXCL1, 2 and 3) and CXCL8. These chemokines, primarily through the receptors CXCR1 and CXCR2, stimulate angiogenesis and respond to the activation of nuclear factor-*κ*B (NF-*κ*B), a major mediator of inflammation [8, 25]. 

CXCL8 is one of the dominant transcriptional targets of the inflammatory signaling mediated by nuclear factor-*κ*B (NF-*κ*B), which is commonly activated in cancer cells. CXCL8 is a proinflammatory chemokine that acts on leukocytes and endothelial cells, via their CXCR1 and CXCR2 receptors, to promote immune infiltration and angiogenesis, which in turn establishes a venue for cancer cell local invasion, migration, and metastasis [26]. As an angiogenic chemokine, CXCL8 binds with high affinity to both the CXCR1 and CXCR2 receptors, contributing to its function in the cancer microenvironment. The present review focuses on the function of CXCL8, as it relates to oncogenic processes and to drug response.

## 3. CXCL8 as a Chemokine

CXCL8, also known as interleukin 8 (IL-8), is a CXC-type chemokine originally identified as a leukocyte chemoattractant [27, 28]. The CXCL8 gene encodes for a precursor protein of 99 amino acids, which, upon processing, yields active proteins of either 77 amino acids in nonimmune cells or 72 amino acids in monocytes and macrophages [29, 30]. CXCL8 signals through CXCR1 and CXCR2 G-protein-coupled receptors [31, 32]. 

In the context of tumors, an essential effect of CXCL8 is its initiation of leucocyte infiltration and neovascularization, which precede invasion and metastasis. This tumor progression may occur as a function of the regulation of angiogenesis, cell motility, immune cell infiltration, cell growth and survival in the microenvironment, and modulation of local antitumor immune responses. CXCL8 enhances the proliferation and survival of endothelial cells and up-regulates the expression of two matrix metalloproteinases, MMP-2 and MMP-9 [33–35]. CXCL8 also mimics the function of vascular endothelial growth factor (VEGF), trans-activates VEGF-R2, and promotes angiogenesis [36]. In cancer models of the liver, pancreas, colorectum, and melanoma, CXCL8 functions as an autocrine growth factor [26, 37–41].

This evidence indicates that CXCL8, produced in an inflammatory microenvironment, aggravates the inflammatory state and enables cancer cells to survive and to migrate from the primary site.

## 4. Regulation of CXCL8 Expression

Research into the role of CXCL8 in cancer has been hampered by the lack of a homologous gene in the mouse, the common animal model for studies of human cancer, and by the functional overlap between various chemokines [27]. Therefore, the knowledge about the CXCL8 gene and its regulation is derived mostly from cultured or isolated cells. For the same reasons, translational application of knowledge of CXCL8 biology is also complicated. The most clinically relevant human data on CXCL8 refer to the correlation between high serum levels of the chemokine and poor prognoses, [42, 43] suggesting that patients expressing high levels of inflammatory cytokines or affected by inflammatory types of cancers are at a high risk of having aggressive cancers. Cancers associated with inflammation generally display the characteristics of aggressiveness [15, 44]. Since CXCL8 is not the only chemokine expressed under such circumstances, determination of its specific role is difficult.

Various signals and/or pathways induce CXCL8 expression in cancers [40]. The activation of oncogenes may be linked to inflammatory signaling via the cell-intrinsic mechanism described above. For instance, the RAS-RAF signaling pathway activates the NF-*κ*B transcription factor, which in turn leads to the production of numerous cytokines [45]. Some of these cytokines (e.g., CXCL8 and IL-6) are proinflammatory, and their continued production supports the transformation of cells into malignancy and invasiveness [46–48]. Moreover, there is persistent NF-*κ*B activation in cancers associated with chronic inflammation [48–52]. Tumor angiogenesis, growth, and metastasis are also facilitated by NF-*κ*B-induced transcription of genes for cytokines and other proteins. In addition to NF-*κ*B, activator protein-1 (AP1) also regulates the expression of CXCL8 [53], and there is a possible involvement of EGFR signaling in the regulation of CXCL8 production/expression [54].

Once expression of CXCL8 has been induced, this chemokine may also feed forward to activate NF-*κ*B and to exacerbate the inflammatory cycle [55]. For example, there is a correlation between CXCL8 expression and growth, angiogenesis, and metastasis of colon carcinoma cells after activation of NF-*κ*B [56]. Although cytokine-mediated activation of NF-*κ*B is the main mechanism for transcriptional induction of chemokines, NF-*κ*B may cooperate with other pathways in this process. The transcription of *CXCL8 *is modulated mainly through an NF-*κ*B response element that works in concert with adjacent AP1 and elements of nuclear factor induced by IL-6 (NF-IL-6) [30, 57].

## 5. CXCL8 Signaling

**Figure 1:**
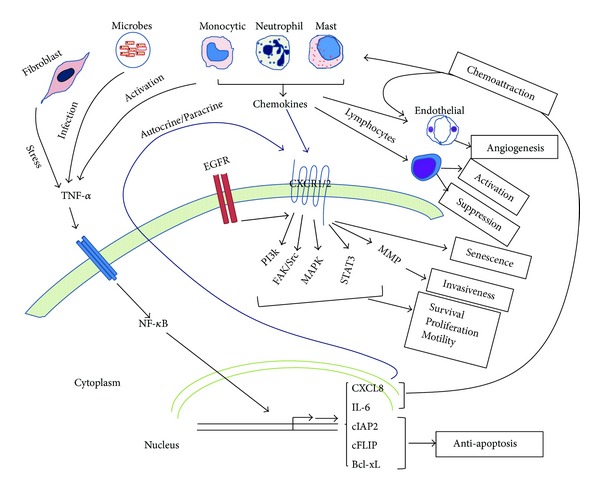
Principal mechanisms of CXCL8 regulation and signaling. NF-*κ*B activation is initiated primarily by TNF*α* released by stressed fibroblasts, in response to chronic infection, or by activated leukocytes (monocytes, neutrophils, and mast cells). NF-*κ*B is the primary regulator of the chemokines CXCL8 and IL-6, which are potent chemoattractants for leukocytes, especially neutrophils. Other major transcriptional targets of NF-*κ*B include the anti-apoptosis proteins, cIAP2, cFLIP, and Bcl-xL. CXCL8 signals through CXCL1 or CXCL2, whereas IL-6 signals through the IL-6 receptor (IL-6R). Leukocytes attracted to the initiated tumor secrete cytokines that drive the tumorigenic process by promoting angiogenesis through endothelial cell proliferation and modulation of lymphocyte responses. CXCL8 directly activates endothelial cells through their CXCR1 or CXCR2 receptors. CXCL8 binds to CXCR1 and CXCR2, and, in cooperation with EGFR signaling, may promote cancer cell survival, proliferation, motility, and invasiveness through the PI3K, MAPK, FAK/Src, STAT3, or MMP pathways. Since tumor cells may also express CXCR1 or CXCR2, CXCL8, in the tumor microenvironment, may signal through both paracrine and autocrine mechanisms.

CXCL8 signals through CXCR1 and CXCR2, receptors present in various types of normal as well as cancerous cells. These are targets for autocrine and paracrine signaling by CXCL8 and other chemokines that use these receptors [3, 58]. Since CXCR1 and CXCR2 receptors are expressed on cancer cells, endothelial cells, neutrophils, and tumor-associated macrophages, the synthesis and secretion of CXCL8 from tumor cells affects the tumor microenvironment [8]. Hence, CXCL8 signaling is involved in regulating the communication between these cell types within the tumor microenvironment (Figure 1). Despite the dominant effect of CXCL8, the dynamics of chemokine release and activity in the tumor microenvironment are complex, and the balance between CXCL8 and other cytokines should be considered. Again, analogous to the scenario of exposure to a foreign pathogen or antigen, the success or failure of antitumor immunity could be determined by the net balance of the effector cytokines in the microenvironment.

 Phosphtidylinositol-3 (PI3) is a component in CXCR1/2-signaling. The enzyme PI3-kinase (PI3K) is a principal effector of CXCL8-mediated chemotaxis in neutrophils. This increased phosphorylation results in the activation and increased expression of the serine/threonine kinase, PKB/Akt [59–61]. CXCL8 may also regulate the activity of the mitogen-activated protein kinase (MAPK) cascade in ovarian cancers, where there is a crosstalk with the EGFR pathway through the activation of CXCR1/2 [62]. Similarly, CXCL8 activates the classical MAPK signaling cascade, with downstream phosphorylation of Erk1/2 in neutrophils and cancer cells [63]. Activation of MAPK signaling is consistent with the promotion, by IL-8, of proliferation and survival for various types of cells [63, 64]. The classical cascade between Erk and MAPK signaling describes a pathway linking CXCL8 to the activation of E2F and activator protein transcription factors, the main function of which is to regulate the transcription of genes associated with cell proliferation [17, 46, 65]. 

 In endothelial and cancer cells, protein tyrosine kinases are farther downstream in the IL-8 signaling pathway. Additionally, CXCL8 induces the activation of VEGFR-2 in endothelial cells [66]. Focal adhesion kinase (FAK) and Src-kinases are also activated in cancer cells stimulated with CXCL8 [67]. Activation of Src and FAK signaling is consistent with increases in cellular proliferation, survival, and chemoresistance, and with regulation of cell spreading, motility, and invasion [68]. As a result of multiple pathways being triggered in response to CXCL8 signaling, transcription factors may be activated in cells exposed to this chemokine. 

Signaling through CXCR2 leads to senescence, especially in p53-proficient and nontransformed cells [69]. Since CXCR2 has multiple ligands; however, the specific contribution of CXCL8 to senescence has not been established. In contrast, a role for IL-6, another proinflammatory cytokine, in senescence and in relation to the senescence-associated secretory phenotype has been established [47, 70]. The principal mechanisms of CXCL8 regulation and signaling are summarized in Figure 1. 

## 6. Role of CXCL8 in Cancer Progression and Metastasis

In addition to the lack of a homologous genetic model, efforts to determine the function of CXCL8 in cancer progression are complicated by redundancy of the chemokines that share CXCR1 and CXCR2, and by the expression of cytokines other than CXCL8 in response to an upstream stimulus. Even in the absence of CXCL8, chemokines such as CXCL1 and CXCL6 would still attract immune cells to the “inflamed” site [8]. CXCL8 promotes cancer cell proliferation, survival, and migration via its autocrine and paracrine activity, and it elicits an angiogenic response in endothelial cells and chemotaxis of neutrophils to the tumor site via its paracrine activity [6, 71–73]. Since, in the microenvironment, tumor cells are surrounded by fibroblasts, dendritic cells, tumor-associated macrophages, and other cells of lymphoid origin; CXCL8 produced by tumor cells could act on one or more of these cells, producing other cytokines, growth factors, and/or MMPs. In addition to the local effects of these chemokines, metastasis of cancer cells is facilitated by CXCL8 and its receptors on tumor cells, which enables them to undergo the epithelial-mesenchymal transition, and then to migrate and seed at secondary sites [3, 54, 74–77]. Moreover, in response to stress, stromal cells produce CXCL8, which may influence the invasiveness and/or metastatic potential of cancer cells [78, 79]. 

 Chemokines and their receptors are involved in directing organ-specific metastasis to regional lymph nodes and to other sites where the ligands are expressed [25, 80]. As stated above, such migration of cancer cells is analogous to the migration of antigen-presenting cells from their sites of normal residence. Under the classical response to a localized infection, professional antigen-presenting cells (APCs) process and present antigenic epitopes to effector cells, characteristically through a process that includes an APC chemokine response, epithelial-mesenchymal-transition, and migration to the draining/local lymphoid tissues [81–83]. In doing so, the APCs mount and coordinate a defense against a foreign pathogen. 

Consistent with this analogy, once recruited into the tumor, infiltrating APCs would be expected to coordinate the immune response against malignant cells. It could also be anticipated that, in the absence of a foreign antigen signature (a pathogen-associated molecular pattern), the action of recruited APCs would be to recruit suppressor regulatory immune cells and to subdue any anticancer activity of effector cells in the tumor microenvironment, a process analogous to the “resolution phase” after control of a pathogen-associated inflammatory reaction [84]. Under this circumstance, owing to the permissive microenvironment and to the loss of sensitivity to environmental cues that normally restrict unregulated proliferation of cells, malignant cells would continue to expand, invade, and emulate the migratory (metastatic) path of professional APCs to regional and distant sites. 

Regarding the role of CXCL8, a question is whether or not primary cancer cells and their metastatic derivatives differ in their expression and secretion of CXCL8. Other questions are: (a) if the primary cancer cells utilize CXCL8- (and/or other cytokine) mediated mechanisms to migrate and establish a metastatic site, would further expression of these chemokines at the new site be essential? (b) Are metastatic sites equally infiltrated by leukocytes? (c) What is the frequency of secondary metastasis from the first metastatic site? Answers to these questions could help us understand if therapeutic approaches targeted to cytokines would be beneficial against a cancer that has already metastasized.

## 7. CXCL8 and Drug Response

Multiple reports indicate that chemokines and their receptors are valid targets for new therapeutic agents against cancer. An evident challenge in this approach is the chemokine response to chemotherapeutic drugs and radiation therapies. 

During cancer therapy, NF-*κ*B signaling is involved in orchestrating chemokine responses. In addition to being aberrantly activated in cancer cells, NF-*κ*B is activated by most modalities of cancer therapy [85–87], and aberrant activation of NF-*κ*B is proposed as a major factor contributing to the resistance to chemotherapy. Studies conducted with cultured cells show that inhibition of NF-*κ*B, by drugs or natural compounds, sensitizes cells to apoptosis through inhibition of the expression of antiapoptotic genes [88, 89]. 

 Evaluated in cancer patients, CXCL8 expression might be used to assess the patient's prognosis and response to chemotherapy. In various types of human cancers, high levels of CXCL8 in serum or at local sites correlate with aggressive disease and poor initial response to drugs, including oxaliplatin, 5-fluorouracil, paclitaxel, and camptothecin [53, 90–94]. In contrast, paclitaxel, camptothecin, and erlotinib increase CXCL8 transcription and secretion in cancer cells [95, 96] (and our unpublished data). Thus, the significance of CXCL8 in modulating the response of cancer cells to chemotherapy is still not fully understood. Nevertheless, the potential use of CXCL8 as a diagnostic or prognostic marker has been advocated [97, 98]. 

 The concept of developing treatment strategies to alter the tumor microenvironment or to interrupt interactions between cancer cells and their environment is gaining momentum [99]. Nevertheless, there are questions that remain unanswered regarding the complex chemokine system. For example, direct targeting of NF-*κ*B components, NF-*κ*B transcriptional targets, and antagonizing chemokine receptors or downstream signaling seem to be attractive strategies to increase the effectiveness of chemotherapy and radiation. Since inflammatory mechanisms that are associated with chronic infections, autoimmune diseases, and cancer overlap functionally, knowledge gained from these diseases should be assimilated. Indeed, some CXCR1 and 2 antagonists, initially developed for inflammatory diseases, are currently under consideration for or actually in clinical trials for cancer therapy (http://clinicaltrials.gov/) [100–104].

## 8. Conclusion

The chemokine and chemokine receptor signaling networks are important not only from immunological perspectives but also as factors in cancer progression and metastasis and as modulators of responses to chemotherapy and radiation. CXCL8, a cytokine induced by activated NF-*κ*B signaling, appears to be involved in these mechanisms. Further studies will reveal how such information can be used to develop new strategies to prevent or treat cancer. Just as the outcome of an infection or vaccination is determined by the balance of chemokine responses, the fate of a tumor, whether it stays benign and for how long, whether it spreads and how fast and where to, whether it is rejected or accepted by immune cells, and whether a given therapeutic agent eliminates or exacerbates its growth, may be determined by the dominance of certain chemokines, their receptors, and signaling partners in cells in the tumor microenvironment. In this respect, the regulators, receptors, signaling pathways, and effectors of chemokines such as CXCL8 provide attractive targets for cancer therapeutic intervention. Furthermore, serum or tissue levels of CXCL8 and its receptors could prove to be useful as biomarkers for prognosis, drug efficacy, and/or drug responses.
